# Higher baseline resting metabolic rate is associated with 1-year frailty decline among older adults residing in an urban area

**DOI:** 10.1186/s12877-023-04534-5

**Published:** 2023-12-07

**Authors:** A Gonzalez, J Soto, N Babiker, K Wroblewski, S Sawicki, D Schoeller, A Luke, Megan Huisingh-Scheetz

**Affiliations:** 1 University of Chicago, Chicago, USA; 2 Illinois Institute of Technology, Chicago, USA; 3 Department of Public Health Sciences, University of Chicago, Chicago, USA; 4 Department of Medicine, Section of Geriatrics and Palliative Medicine, University of Chicago, Chicago, USA; 5 University of Wisconsin in Madison, Madison, USA; 6 Department of Public Health Sciences, Loyola University, Chicago, USA

## Abstract

**Background:**

Dysregulated energy metabolism is one hypothesized mechanism underlying frailty. Resting energy expenditure, as reflected by resting metabolic rate (RMR), makes up the largest component of total energy expenditure. Prior work relating RMR to frailty has largely been done in cross section with mixed results. We investigated whether and how RMR related to 1-year frailty change while adjusting for body composition.

**Methods:**

N = 116 urban, predominantly African-American older adults were recruited between 2011 and 2019. One-year frailty phenotype (0–5) was regressed on baseline RMR, frailty phenotype, demographics and body composition (DEXA) in an ordinal logistic regression model. Multimorbidity (Charlson comorbidity scale, polypharmacy) and cognitive function (Montreal Cognitive Assessment) were separately added to the model to assess for change to the RMR-frailty relationship. The model was then stratified by baseline frailty status (non-frail, pre-frail) to explore differential RMR effects across frailty.

**Results:**

Higher baseline RMR was associated with worse 1-year frailty (odds ratio = 1.006 for each kcal/day, *p* = 0.001) independent of baseline frailty, demographics, and body composition. Lower fat-free mass (odds ratio = 0.88 per kg mass, *p* = 0.008) was independently associated with worse 1-year frailty scores. Neither multimorbidity nor cognitive function altered these relationships. The associations between worse 1-year frailty and higher baseline RMR (odds ratio = 1.009, *p* < 0.001) and lower baseline fat-free mass (odds ratio = 0.81, *p* = 0.006) were strongest among those who were pre-frail at baseline.

**Discussion:**

We are among the first to relate RMR to 1-year change in frailty scores. Those with higher baseline RMR and lower fat-free mass had worse 1-year frailty scores, but these relationships were strongest among adults who were pre-frail at baseline. These relationships were not explained by chronic disease or impaired cognition. These results provide new evidence suggesting higher resting energy expenditure is associated with accelerate frailty decline.

**Supplementary Information:**

The online version contains supplementary material available at 10.1186/s12877-023-04534-5.

## Introduction

Frailty is a medical syndrome defined as increased vulnerability to adverse health outcomes [1]. Frailty is characterized by a decrease in physiological reserve [2] and can be diagnosed using one of several available tools [3, 4]. The most commonly used tool is the frailty phenotype which is based on a biological model of frailty and evaluates impairments in five areas: weakness, slowness, low physical activity, exhaustion, and unintentional weight loss [1, 5]. These domains were selected to represent the various physiological disturbances hypothesized to drive frailty’s pathophysiology. Core elements central to this proposed biological mechanism include (1) decreased total energy expenditure through a reduction of elective physical activity and decreased resting metabolic rate resulting from sarcopenia and (2) unintentional weight loss due to chronic undernutrition and a net negative energy balance, despite lower total energy expenditure. Frailty is associated with an increased risk of major surgical complications, hospital readmission, length of hospital stay, and risk of death [6–10]. Due to the substantial impact of frailty on older adult outcomes, it is critical that we better understand the energetic relationships that underlie frailty decline, particularly as it may help identify frailty mitigation targets.

Total energy expenditure (TEE) is composed of three main energetic costs: activity energy expenditure (AEE), resting metabolic rate (RMR), and the thermic effect of food. AEE is further divided into volitional exercise and non-exercise activity thermogenesis (NEAT) [11]. Volitional exercise and NEAT are highly variable energetic costs and combined can comprise anywhere from 20 to 50% of TEE [11]. RMR accounts for 60–80% of TEE, and the thermic effect of meals comprises a small 10% fraction of TEE [11]. The large majority of prior literature studying energetics and frailty has focused on physical activity or physical activity energy expenditure. Low physical activity is both an indicator of frailty and an accelerator of frailty decline. In cross-section, pre-frail and frail adults have lower daytime physical activity levels than non-frail adults, particularly in the morning [12, 13]. In a cross-lagged panel analysis over two years by Sagong et al., frailty and high physical activity had a significant reciprocal relationship in a middle-aged group (70–79 years) where high physical activity predicted less frailty after two years [14]. In the oldest group, however, there was no statistically significant relationship between frailty and physical activity. Similarly, non-frail adults reporting more moderate to vigorous physical activity participation experienced slower progression to frailty over five years [15]. RMR has been the focus of substantially less frailty work to date even though it is the largest component of TEE.

The limited work exploring the relationship between RMR and frailty has largely been done in cross-section. In a 2019 cross-sectional study, Bastone et al. used the frailty phenotype to assess frailty status, doubly-labeled water to measure TEE, and indirect calorimetry to measure RMR, and found that RMR was not different between frail and non-frail adults [16]. In contrast, Abizanda et al. found that frail and pre-frail older adults (based on frailty phenotype criteria) have lower RMR compared to their non-frail counterparts by an average of 114 and 160 kilocalories/day, respectively, after adjusting for fat-free mass (FFM) but not fat mass (FM) [17]. To our knowledge, there are no longitudinal studies evaluating the relationship between RMR and change in frailty to better understand how resting energetic demands relate to frailty risk over time.

Various factors are known to affect RMR in older adults including sex, body composition, and multimorbidity. RMR is heavily influenced by lean body mass (i.e., fat-free mass), the most metabolically active tissue in the body; RMR declines are closely associated with loss of lean mass [18]. Females tend to have a lower RMR than males, independent of differences between body composition and aerobic fitness [19]. RMR is altered by chronic diseases though this relationship may be dynamic over time. In a 10-year longitudinal study, multimorbid women experienced an increase in RMR compared to those without multimorbidity, independent of age and body composition [20]. In a 13-year study, cancer and diabetes were associated with higher RMR at baseline and COPD, cancer, diabetes, heart failure, and chronic kidney disease were associated with greater declines in RMR than individuals without these conditions [21]. Frail adults tend to have baseline differences in body composition including lower muscle mass, lower bone mass, and a higher fat percentage [22]. A cross-sectional study of participants in the Baltimore Longitudinal Study on Aging, adults (40–96 years) who were free from cognitive and physical impairments, chronic conditions, and blood test alterations had a 109.6 kcals/day lower RMR relative to participants with any of the impairments [23]. Therefore, studying the RMR and frailty relationship cannot be done without accounting for sex, body composition and multimorbidity.

The limited and conflicting data on the relationship between RMR and frailty warrants further analysis. The objectives of this study were to: (1) relate objectively measured resting metabolic rate and body composition to one-year change in frailty among predominantly African-American older adults, (2) examine the effects of adjusting for multimorbidity (and separately cognition) on this relationship, and (3) explore any differences in the RMR-frailty relationship across different levels of frailty at baseline.

## Methods

### Study population

Study participants (n = 151) were recruited between July 2011 and October 2019 from the community residing around the primary geriatrics practice site for the University of Chicago located on the south side of Chicago, a community with a high proportion of minority older adults. The sample was limited to community-dwelling older adults, who were 65 or older. Exclusion criteria included hospitalization or surgery/procedure within two months of participating in the study; addition or change in dose of thyroid (e.g. levothyroxine) or a diuretic (e.g. furosemide, hydrochlorothiazide, spironolactone) medication within two months of participating in the study; use of oral steroids; use of beta blocker (e.g. metoprolol, atenolol, carvedilol); persistent hyperglycemia greater than 250; life expectancy less than one year (because outcome data were collected at 1 year); and history of moderate or advanced dementia, a Montreal Cognitive Assessment score ≤ 18 at baseline or an inability to understand the consent using a Teach-Back method [24]. Hospital, surgery, medication, and hyperglycemia exclusion criteria were required for ideal RMR testing at baseline. Data collection occurred over multiple evaluations: (1) baseline survey and physical exam, (2) a baseline seven-day free-living hip accelerometry protocol, (3) baseline fasting resting metabolic rate measurement with indirect calorimetry and DEXA scan for body composition within two weeks of baseline survey and physical exam, (4) a one-year follow-up survey and physical exam. This study protocol was reviewed and approved by the University of Chicago Institutional Review Board (IRB #13–0443). All study participants enrolled in this study underwent written informed consent. Our final analytic sample for the current study included n = 116 participants who had non-missing data for all independent and dependent variables (excluding polypharmacy and income for which we included a missing category) at baseline and 1 year. Four additional participants had non-missing baseline data but had delayed 1-year frailty assessments until 2-years (n = 2) or 3-years (n = 2). We conducted sensitivity analyses including the n = 4 individuals with the n = 116 (total n = 120) using the later frailty data as a surrogate for 1-year frailty.

The sample characteristics of the n = 35 excluded participants (Supplemental Table 1) were compared to the included sample using Wilcoxon rank-sum tests for continuous/ordinal variables and chi-square tests for categorical variables. The excluded sample did not significantly differ from the included participants by baseline RMR, body composition, race, gender, chronic medical condition burden or polypharmacy variables but they were significantly older (mean age 76.0 versus 72.8, *p* = 0.008), less educated (< high school education 17.1% versus 2.6%, *p* = 0.001) and had significantly lower cognitive function (Montreal Cognitive Assessment mean 23.5 versus 25.6, *p* = 0.001). While the mean baseline frailty scores among those excluded were not significantly different from the included sample (1.4 versus 1.0, *p* = 0.08), the proportion of non-frail, pre-frail, frail and missing were significantly different (20.0% versus 39.7% non-frail, 51.4% versus 53.5% pre-frail, 20.0% versus 6.9% frail, 8.6% versus 0% missing, *p* = 0.001). While the excluded sample had higher income proportionally, ($0-<2000/month 42.9% versus 47.4%, $2000–3999/month 20.0% versus 31.0%, $4000–5999/month 28.6% versus 9.5%, $6000/month 8.6% versus 6.9%, missing 0 versus 6, *p* = 0.04), there were more participants with missing data among the included sample. Among the n = 35 excluded for missing data, 20 of our original n = 151 (13.3%) experienced attrition for various reasons, n = 11 were missing RMR data, and n = 11 were missing body composition data (Supplemental Table 1).

### Resting metabolic rate

Resting metabolic rate (RMR) was measured in mean kilocalories (kcal) / day using indirect calorimetry (Sensormedics Vmax Encore 29n Nutritional Assessment Instrument, Yorba Linda, CA). Following an overnight 12-hour food and alcohol fast, participants traveled to the medical center, and rested on a bed for 20 min prior to beginning measurement, with the bed at a 25 degree angle. Respiratory gas exchange was measured by certified staff for at least 45 min in a quiet, dimly lit room. Blankets were provided for comfort. Participants were asked to avoid any movement, talking or sleeping during measurement. Intervals during which these activities were observed to occur were excluded. Four indirect calorimetry machines were used throughout this study. These machines underwent cross validation and flow sensor and a gas analyzer calibration before the measurements.

*RMR data processing.* Of the 6603 measurements (one measurement per minute) of RMR testing available in the sample, we excluded partial data from five participants due to restricted activity observations noted by the certified staff: (1) The first 28 measurements were dropped from one participant due to staff noting body movement. (2) The first 35 measurements were dropped for another participant due to variable respiratory quotient readings noted by staff. (3) The entire reading was dropped for another participant due to staff noting body movement and talking throughout the assessment. (4) The entire reading was dropped for another who ate breakfast prior to the appointment. (5) The entire reading was dropped for one participant who had only 17 min of data. After these changes, 6432 min of data remained.

The RMR data then underwent additional manual processing prior to summarizing the data. The first ten measurements (e.g., the first ten minutes of recording) were excluded for all participants (1364 measurements) to provide time for participants to settle into resting state leaving 5,068 measurements. Measurements < 500 kcal/min were excluded given potential apnea, breath holding or movement triggers for such low values (no additional minutes were excluded for this reason). The coefficient of variance (CV) was then calculated for all participants using the remaining data. For individuals with CVs > = 10%, outlier data were excluded until the CV was < 10% [25]. After outlier exclusion (53 measurements, ~ 1% of the data), 5,015 measurements remained. Remaining data were averaged to estimate a RMR in kcal/day for each participant.

### Body composition

Body composition was assessed by dual-energy X-ray absorptiometry (DEXA, Lunar Prodigy GE Healthcare, reference number: DF + 350,660). A technologist certified in Lunar DEXA imaging performed all scans. For our analysis, we extracted the following variables: fat-free mass (kg) and fat mass (kg). Both were treated as continuous variables.

### Physical frailty

Physical frailty was the primary outcome, and it was measured using an adapted frailty phenotype score (Supplemental Table 2) [1]. Unintentional weight loss was identified when the difference in measured baseline weight and self-reported weight 1 year prior was greater than or equal to 5% of body weight or ten pounds. Weakness was identified when the average of 3 dominant grip strength measurements (Jamar hydraulic hand dynamometer) was below the established body mass index- and gender-adjusted cut-points, as previously described [1]. Slowness was identified when the average of three 15-foot usual walk times were below gender- and height-adjusted cut points, as originally described [1]. Exhaustion was identified through a survey tool when participants either reported that they “felt that everything was an effort” or that they “could not get going” 3–4 days (or a moderate amount of the time) or more in the prior 1 week (response options: None of the time, Some or little of the time (1–2 days), A moderate amount of the time (3–4 days), or Most of the time (> 4 days)). Finally, low activity was identified when the kcal/week calculated using responses to the 6-item Minnesota Leisure Time Physical Activity Questionnaire were below gender-adjusted thresholds, as previously described [26]. A point was assigned for each criteria met. The total score ranged from 0 to 5: 0 criteria indicated a non-frail status, 1 to 2 criteria indicated a pre-frail status, and 3 or more indicated a frail status. Physical frailty was measured at baseline and 1 year.

### Covariates

Covariates were collected at baseline and included demographic and health conditions. *Age* was calculated using date of birth and survey date and treated as a continuous variable. Study participants self-identified *race (*African-American versus non African-American); *gender* (woman versus man); *education* (< high school versus ≥ high school); and *monthly individual income* (<$2000/month, $2000–3999/month, $4000–5999/month, $6000+, or Missing). The *Charlson comorbidity index* was calculated using self-reported comorbidity data [27]. *Polypharmacy* was assessed by summing the number of reported medications and then dichotomizing the variable at ≥ 5, < 5 medications or Missing. The Montreal Cognitive Assessment (MoCA) was administered to all participants to evaluate *cognitive function* [28]. MoCA scores range from 0 to 30, with higher scores indicating better cognitive function. It was treated as a continuous variable. For participants with missing covariate data (only income and polypharmacy), a separate ‘missing’ category was created, facilitating the inclusion of these participants in the models.

### Statistical analysis

Sample characteristics were generated for each frailty subgroup: non-frail, pre-frail, frail. Means and standard deviations (SD) were reported for continuous variables and number of participants in each category were reported for categorical data. We identified significant differences across frailty categories using Kruskal-Wallis tests (continuous measures) or chi-squared tests (categorical measures). We also described the frequency of frailty category (non-frail, pre-frail, frail) shifts at one year in the sample.

We then regressed the 1-year frailty phenotype scores (0–5) on baseline RMR, baseline frailty phenotype scores (0–5), body composition (both fat mass and fat-free mass, continuous) and demographics in an ordinal logistic regression model. We then separately adjusted for (1) comorbidities and polypharmacy and then (2) cognition, examining any changes in the RMR-frailty relationship. We adjusted separately for chronic disease burden and cognitive function for three reasons: (1) the Charlson Comorbidity Index includes self-reported dementia, (2) our sample size was small so we hoped to reduce the potential for overfitting the model and (3) we were interested in assessing the impact of each of these factors independently. Output are reported as odds ratios for each model. The model was then stratified by baseline frailty status (non-frail, pre-frail) to explore differential RMR effects across the frailty spectrum.

We conducted additional sensitivity analyses. We reran our primary ordinal logistic regression models using the n = 120 that included n = 4 with delayed frailty follow-up data collection. We then also categorized RMR into quartiles (< 1145 kcal/day, 1145 to < 1279 kcal/day, 1279 to < 1391 kcal/day, and 1391 + kcal/day) and regressed 1-year frailty on the categorical RMR, baseline frailty, body composition and demographics in an ordinal regression model. We further reran our ordinal logistic regression models including self-reported physical activity energy expenditure as a covariate. We used average kcal/week as a continuous variable calculated using responses to the 6-item Minnesota Leisure Time Physical Activity Questionnaire [26]. We then also created a dichotomous outcome variable classifying participants as having “worse” frailty at 1-year versus “stable/better” frailty at 1-year compared to baseline. We regressed this dichotomous variable on baseline frailty, RMR, body composition and demographics using logistic regression, separately among those who were non-frail and pre-frail at baseline.

The within-person measurement reliability for RMR in older adults is unknown. Among 40 overweight, young- to middle-aged adults ages 21–40 years, the two-week within-person reliability was good (mean difference between measurements was 17.4 kcal/day, 95% CI -4.9 to 39.7 kcal/day) [29]. Internal communication with the authors of this paper reported the *R*^*2*^ between the two measures was > 0.9. Due to the potential for measurement error in RMR, we performed a robustness check of our findings. We replicated the models with linear regression and errors-in-variables regression, the latter accounting for an RMR reliability of 0.9. The beta coefficients and significance of the RMR independent variable were largely unchanged (data not shown).

## Results

### Sample characteristics

Table 1 summarizes the sample characteristics by baseline frailty status: n = 46 were non-frail, n = 62 were pre-frail, and n = 8 were frail. Pre-frail and frail adults included proportionally more African-Americans and had a higher comorbidity burden than non-frail adults. The three groups did not have statistically significant differences in age, gender, education, income, polypharmacy, MoCA scores, body composition or RMR.

**Table 1:** Sample Characteristics (Baseline, n = 116)

	Non Frail (n = 46)	Pre Frail (n = 62)	Frail (n = 8)	*p*-value^1^
Age (mean, SD, median, IQR)	72.0 (5.8, 70.59, 67.5–75.5)	73.4 (5.9, 71.4, 69.6–76.5)	72.9 (3.6, 75.3, 70.2–75.9)	0.25
Female (n)	37	51	7	0.89
<High School (n)	1	2	0	0.84
African-American (n)	32	55	8	**0.02**
Monthly Individual Income (n)				
<$2000/month $2000–3999/month $4000–5999/month $6000+ Missing	19 12 3 7 5	32 21 7 1 1	4 3 1 0 0	0.07
Charlson Score (mean, SD, median, IQR)	0.5 (0.9, 0, 0–1)	1.3 (1.6, 1, 0–2)	2.3 (1.2, 2, 2–3)	**0.002**
Polypharmacy (n)				
< 5 meds ≥ 5 meds Missing	35 10 1	35 23 4	4 4 0	0.19
Montreal Cognitive Assessment (mean, SD, median, IQR)	26.0 (2.3, 26, 25–28)	25.5 (2.7, 26, 24–27)	24.5 (2.9, 25.5, 22-26.5)	0.42
Body Composition:				
Fat-Free mass (%, mean, SD, median, IQR) Fat-free mass (kg, mean, SD, median, IQR) Fat mass (kg, mean, SD, median, IQR)	58.0 (7.8, 58.0, 53.1–63.7) 45.4 (9.6, 43.1, 37.8–50.9) 33.4 (12.6, 31.6, 25.2–38.2)	57.3 (9.1, 55.1, 50.3–62.0) 44.0 (7.9, 42.6, 39.1–46.8) 33.7 (10.5, 34.2, 27.2–39.7)	55.2 (10.6, 52.6, 46.8–62.4) 47.9 (8.7, 45.7, 41.3–51.1) 42.1 (19.3, 39.8, 25.9–61.0)	0.43 0.44 0.52
Resting Metabolic Rate (kcal/day, mean, SD, median IQR)	1292 (205, 1285, 1135–1392)	1262 (200, 1285, 1144–1391)	1298 (170, 1215, 1173–1419)	0.77

^1^*p*-values from Kruskal-Wallis test (continuous measures) or chi-square test (categorical measures)

### 1-year frailty transitions

**Fig. 1:**
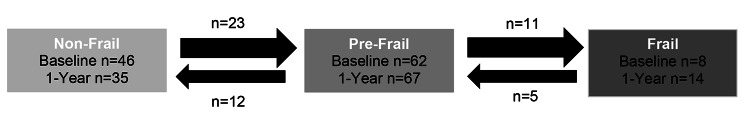
1-Year Frailty Transitions. One-year frailty categorical transitions among n = 116 urban, predominantly African-American older adults. Frailty was measured using the frailty phenotype criteria (range 0–5) and categorized as follows: non-frail = 0 criteria met; pre-frail = 1–2 criteria met; frail = 3 + criteria met

A substantial proportion of study participants transitioned between states of frailty, even across 1 year (Fig. 1). Thirty-four participants progressed to a worse state of frailty: 23/46 non-frail participants transitioned into pre-frail status and 11/62 pre-frail participants transitioned into frail status. Seventeen transitioned to an improved frailty state at 1 year: 5/8 frail participants transitioned into a pre-frail status, 12/62 pre-frail participants transitioned into a non-frail status.

### Ordinal logistic regression model relating rmr to 1-year frailty

The multivariate ordinal logistic regression models associating baseline RMR to 1-year change in frailty are shown in Table 2. In a model adjusting for baseline frailty, body composition and demographic covariates, higher RMR at baseline was significantly associated with higher (worse) frailty scores at 1 year (Odds ratio = 1.006 for each kcal/day, *p* = 0.001). Lower fat-free mass was independently associated with higher (worse) frailty scores at 1 year (Odds ratio = 0.88 for each kg increase in mass, *p* = 0.008). Adjusting for comorbidity and polypharmacy burden did not substantially change the relationship between RMR and 1-year frailty (Odds ratio = 1.006, *p* = 0.001) nor did adjusting for cognitive function (Odds Ratio = 1.006, *p* = 0.001). These results also did not change when including the n = 4 participants who had delayed follow-up frailty assessments (data not shown). The model including RMR categorized into quartiles is shown in Supplemental Table 3. The odds ratio for each increasing quartile was associated with a greater risk of 1-year frailty decline after adjusting for demographics: lowest quartile = ref, second quartile OR = 2.55 (*p* = 0.10), third quartile = 3.25 (*p* = 0.07), highest quartile = 6.42 (*p* = 0.02). The odds ratios for the body composition measures were in the same direction and were of similar effect size to the primary model; however, the statistical significance of the fat-free mass covariate weakened (OR = 0.92, *p* = 0.06) and the statistical significance of the fat mass covariate became stronger (OR = 1.05, *p* = 0.05). Adjusting for baseline self-reported physical activity energy expenditure did not greatly affect the relationship between RMR and 1-year frailty in any of the models (Supplemental Table 4).

**Table 2:** Ordinal Logistic Regression Model Relating 1-Year Frailty Phenotype to Baseline Resting Metabolic Rate (n = 116)

	Model 1	Model 2	Model 3
Independent variables	Odds Ratio, (*p* value)	Odds Ratio, (*p* value)	Odds Ratio, (*p* value)
Resting Metabolic Rate (per 1 kcal/day)	1.006 (0.001)	1.006 (0.001)	1.006 (0.001)
Frailty score (baseline)	3.57 (< 0.001)	3.98 (< 0.001)	3.62 (< 0.001)
Fat-Free Mass (per 1 kg mass)	0.88 (0.008)	0.88 (0.01)	0.88 (0.008)
Fat Mass (per 1 kg mass)	1.04 (0.14)	1.03 (0.24)	1.04 (0.15)
Race			
Black Other	1.01 (0.99) Ref	1.25 (0.72) Ref	1.11 (0.87) Ref
Age (per year)	1.07 (0.08)	1.07 (0.07)	1.07 (0.08)
Gender			
Female Male	0.23 (0.09) Ref	0.20 (0.08) Ref	0.22 (0.09) Ref
Education			
≥High School <High School	0.17 (0.25) Ref	0.18 (0.30) Ref	0.18 (0.28) ref
Monthly Income			
<$2000 $2000–3999 $4000–5999 $6000+ Missing	ref 0.81 (0.62) 0.15 (0.009) 2.85 (0.19) 5.83 (0.11)	ref 0.69 (0.40) 0.11 (0.004) 3.14 (0.16) 4.16 (0.20)	ref 0.82 (0.64) 0.14 (0.007) 2.90 (0.18) 5.28 (0.13)
Charlson Comorbidity Index	--	0.81 (0.16)	--
Polypharmacy			
<5 medications ≧5 medications Missing	--	ref 1.78 (0.20) 1.91 (0.52)	--
Montreal Cognitive Assessment Score	--	--	1.07 (0.34)

### Ordinal logistic regression models stratified by baseline frailty status

The multivariate ordinal logistic regression model adjusting for baseline frailty, body composition and demographic covariates was then stratified by baseline frailty status (Table 3). The strongest associations with RMR and fat-free mass were observed among those who were pre-frail at baseline: higher RMR (Odds ratio = 1.009, *p* < 0.001) and lower fat-free mass (Odds ratio = 0.81, *p* = 0.006) were significantly associated with worse 1-year frailty scores.

**Table 3:** Ordinal Logistic Regression Model Relating 1-Year Frailty Phenotype (0–5) to Baseline RMR in Subgroups Stratified by Baseline Frailty Status (Non-Frail or Pre-Frail)

	Non-Frail at Baseline n = 46	Pre-Frail at Baseline N = 62
Independent Variables	Odds Ratio, (*p* value)	Odds Ratio, (*p* value)
Resting Metabolic Rate (per 1 kcal/day)	1.002 (0.53)	1.009 (< 0.001)
Frailty score (baseline)	-- (0 points)	7.83 (< 0.001) (2 vs. 1 point)
Fat-Free Mass (per 1 kg mass)	0.93 (0.34)	0.81 (0.006)
Fat Mass (per 1 kg mass)	1.06 (0.19)	1.008 (0.81)
Race		
Black Other	0.60 (0.53) Ref	0.34 (0.26) Ref
Age	1.08 (0.17)	1.04 (0.41)
Gender		
Female Male	0.27 (0.44) Ref	0.18 (0.17) Ref
Education^1^		
≥High School <High School	-- --	0.05 (0.09) ref
Monthly Income^1^		
<$2000 $2000–3999 $4000+/month Missing	ref 1.07 (0.93) 1.32 (0.74) 6.76 (0.15)	ref 1.21 (0.75) 0.27 (0.12) 0.05 (0.18)

^1^Among the n = 46 older adults who were non-frail at baseline, only n = 1 had an education < High school, therefore the education variable was not included in the non-frail model. Due to small cell sizes, the $4000–5999/month and $6000+/month income categories were collapsed for both models. These adjustments were made to optimize model fitting and did not substantially change any independent variable effect sizes or level of statistical significance

The sensitivity analysis looking at worse 1-year frailty (versus stable/better) is shown in Supplemental Table 5. RMR was significantly associated with worse frailty at 1-year among those who were pre-frail (Odds ratio = 1.009, *p* = 0.02) but not non-frail at baseline (Odds ratio = 1.003, *p* = 0.32). Fat-free mass was not significantly associated with worse 1-year frailty in the logistic regression models among older adults who were pre-frail (Odds ratio = 0.93, *p* = 0.49) or non-frail (Odds ratio = 0.92, *p* = 0.36) at baseline.

## Discussion

The primary objective of this study was to examine the relationship between RMR and 1-year change in frailty in a sample of predominantly African-American older adults, adjusting for body composition, to extend prior work conducted in cross-section. We found that higher baseline RMR and lower baseline fat-free mass were independently associated with worsened frailty at one year after adjusting for baseline frailty status. Neither multimorbidity nor cognitive function at baseline significantly altered this relationship. Unique to this study, we also explored these relationships in models stratified by baseline frailty status. Higher RMR and lower fat-free mass were most strongly associated with frailty progression among those who were pre-frail at baseline. These results provide new evidence suggesting higher resting energy expenditure is associated with accelerated short-term frailty decline.

Over large intervals of time (e.g., five years), frailty generally progresses in older adults; [30] however, over shorter intervals, individuals may move between states of frailty. In this local sample of predominantly African-American older adults, we noted both progression and improvement of frailty statuses, even at one year. While more people in this study experienced frailty progression, about 15% of adults had improved frailty status at one year. It is feasible that frailty status can change over a period as short as one year, especially following an acute stressor that temporarily worsens frailty, such as a hospitalization or acute illness, but from which one is expected to recover. These detected one-year frailty transitions have two important implications for clinical practice and frailty research. The first is that the frailty literature has not yet determined how frequently frailty should be assessed in clinical practice [31, 32]. Most work relating frailty to morbidity and mortality outcomes relies on a single, baseline measure of frailty; however, early evidence suggests older adult physical function trajectories can be differentiated over as short as 3 times points across two years. The two-year trajectories of function improved prediction of mortality above using just baseline measures [33]. Here, we detect frailty category changes over just one year in a substantial proportion of our sample, indicating repeat testing even at one year may help inform these trajectories. The second implication is that these findings raise a question about frailty measurement reliability. Others have reported measurement variability within subjects and across administrators for several measures of physical function [34–36]. Measurement reliability has not been reported for the frailty phenotype itself. Future work exploring how to differentiate measurement variability from clinically significant changes in frailty status is a major gap to translating frailty to clinical practice and represents a limitation to this study [31].

We are among the first to relate RMR to change in frailty phenotype scores in a longitudinal model. After adjusting for both body composition and comorbid illness, higher RMR at baseline was significantly associated with frailty progression at one year. Prior cross-sectional work relating RMR to frailty status have found both depressed RMR [17]) and elevated RMR [37] were associated with worse frailty. Abizanda et al. found RMR was lower among those who were frail and pre-frail relative to those who were non-frail using a categorical frailty phenotype after adjusting for FFM, age, gender and comorbidities [17]. In contrast, Kim et al. found higher RMR was associated with worse frailty using an adapted accumulated deficits frailty index (FI_34_) but only among the oldest men and women (≥ 90 years) after adjusting for FFM, FM, age, sex, and insulin-like growth factor 1 [37]. The FI_34_ heavily weights comorbidities and disability in the frailty score, many of which have independent associations with higher RMR [38, 39]. At baseline and in cross-section, we found no differences in RMR across the three frailty subgroups even after adjusting for covariates (data not shown), further contributing to the diversity of cross-sectional findings. The variability in the collection of the cross-sectional studies might suggest that summary biomarker measures miss important heterogeneity between individuals, especially when the biomarkers also have dynamic trajectories. Alternatively, these findings might suggest that RMR is a better short-term predictive frailty biomarker than a cross-sectional diagnostic frailty biomarker. Finally, these findings might also suggest that RMR is a dynamic biomarker across the spectrum of frailty contributing to mixed associations in cross-section. Our work builds on this prior cross-sectional work by studying a unidirectional and longitudinal association between baseline RMR and change in frailty over one year. Various comorbid illnesses have been associated with changes in energy expenditure [16, 40, 41]. However, multimorbidity did not explain the positive association between higher baseline RMR and worsening frailty in our exploratory study. Cognitive function may also be related to energy utilization [42]. For instance, higher basal metabolic rate measured by bioimpedence analysis was strongly associated with worse dementia-related brain pathology in the UK Biobank study [42]. RMR has also been found to modulate the effects of caloric restriction on improving cognitive function in non-obese adults [43]. Only adults in the caloric restriction arm who demonstrated *increased* RMR showed improved cognition at 24 months. Adults in the caloric restriction arm who demonstrated reduced RMR had comparable cognitive outcomes to the ad libitum diet arm adults, whether they experienced increased or decreased RMR [43]. However, cognitive status also did not alter the association between RMR and frailty in this exploratory study. While neither multimorbidity nor cognitive function were found to mediate the RMR-to-frailty relationship in our study, they have also both been associated with frailty status in cross-section [44, 45].

Our analysis explored only whether baseline RMR was associated with 1-year change in frailty; however, it is possible that frailty has a bidirectional relationship with RMR. For example, Zampino et al. found that chronic illnesses such as congestive heart failure, chronic kidney disease, chronic obstructive lung disease, and cancer predicted a more rapid decline in RMR with aging in the Baltimore Longitudinal Study on Aging dataset; these declines were not accounted for by the acquired cachexia resulting from these conditions [21]. To date, there are no reported studies relating frailty to change in RMR, a potential topic for future work.

Lower fat-free mass was independently associated with worsening 1-year frailty in the overall sample; however, in models stratified by baseline frailty status, the fat-free mass and 1-year frailty association was strongest among the pre-frail group compared to the non-frail group. While some loss of variable significance can be attributed to the small subsample sizes, these stratified findings do raise the possibility that certain body composition measures are stronger risk factors at different frailty stages. Prior work has shown associations between higher fat mass and frailty (cross-sectional), [46] and lower fat-free mass [47] or a combination of these factors known as ‘sarcopenic obesity’ and frailty (longitudinal) [47]. The DEXA used in our study was not able to differentiate muscle quality but muscle fat infiltration may be additionally related to worse frailty [48]. Our stratified models are a unique contribution to the literature and suggest that low muscle mass is a more important frailty risk factor to target among pre-frail. To date, nutrition interventions targeting frailty, primarily protein supplementation, have provided the same nutrition recommendations regardless of degree of baseline frailty [49–51]. These studies have had mixed results. Results from the current study should be confirmed in larger studies but suggest nutritional targets may need to be tailored to different levels of frailty.

This study was limited to a small sample, especially in the stratified models, that included only community-dwelling older adults living in the South Side of Chicago. While it was a strength in our study to include a high proportion of African Americans, a group historically under-represented in the literature and one that suffers greater frailty burden, our results may not apply to other populations. While we found higher baseline RMR was associated 1-year frailty decline, this association does not imply causality. A strength of this study was the use of objective measures to assess both RMR and body composition. A challenge to the clinical relevance of RMR research is the reliance on costly and time-consuming indirect or direct calorimetry following an overnight fast to measure. The less costly option is to use predictive equations incorporating age, gender, height and weight; however, they tend to be less accurate in older adults, and even less accurate when considering comorbid illness [52–55]. There is currently no clinical mechanism for measuring RMR as a risk factor for frailty during clinical care, which represents a barrier to using this metric regularly to risk-stratify patients. Our body composition was measured by DEXA and not MRI or CT, therefore we could not include muscle quality metrics. Our sample size was too small to assess the effects of individual comorbidities like heart failure or COPD, so we combined them into a comorbidity scale. We only had one RMR data point and baseline and 1-year frailty data points. Ideally, RMR and frailty could be measured at the same time over repeated time points. True frailty trajectory modeling would include at least three total data points, which would have facilitated trajectory modeling like the Markov state transition model.

In summary, we conducted a longitudinal study relating RMR and body composition to 1-year change in frailty among predominantly African-American, community-dwelling older adults. In this group, higher baseline RMR and lower baseline fat-free mass were independently associated with frailty decline at one year. Future work in larger, more racially/ethnically-representative samples should explore the relationship of RMR and frailty with repeated measures over time and with more comprehensive measures of chronic illnesses and consideration for bidirectional associations.

### Electronic supplementary material

Below is the link to the electronic supplementary material.

Supplementary Material 1

## Data Availability

The dataset used in the current study is available from the senior author (Dr. Huisingh-Scheetz, megan.huisingh-scheetz@bsd.uchicago.edu) upon reasonable request and after completing the required institutional data use agreement.
