# Supplementary material for: Higher baseline resting metabolic rate is associated with 1-year frailty decline among older adults residing in an urban area
Source: BMC Geriatr. 2023 Dec 7;23:815. doi: 10.1186/s12877-023-04534-5 (PMC10704798; doi:10.1186/s12877-023-04534-5)
Supplement: Supplementary file 1 — Supplementary Material 1 [file 12877_2023_4534_MOESM1_ESM.docx]

**Supplemental Materials**

**Supplemental Table 1: Sample Characteristics of Participants Excluded for Any Missing Data (Baseline, n=35)**

|  | Total Sample |
| --- | --- |
| Age (mean, SD, median, IQR)  Missing | 76.0 (6.8, 74.6, 69.9-81.1)  0 |
| Female (n)  Male (n)  Missing | 29  6  0 |
| <High School (n)  ≥High School (n)  Missing | 6  28  1 |
| African-American (n)  Other race (n)  Missing | 24  11  0 |
| Monthly Individual Income (n)  <$2000/month  $2000-3999/month  $4000-5999/month  $6000+  Missing | 15  7  10  3  0 excluded (6 missing were included in separate ‘missing’ category in analysis) |
| Charlson Score (mean, SD, median, IQR)  Missing | 0.7 (1.2, 0.0, 0.0-1.0)  0 |
| Polypharmacy (n)  <5 meds  ≥5 meds  Missing | 18  15  2 excluded (5 missing were included in separate ‘missing’ category in analysis) |
| Montreal Cognitive Assessment (mean, SD, median, IQR)  Missing | 23.5 (3.8, 24.0, 22.0-26.0)  0 |
| Body Composition  Fat-Free mass (%, mean, SD, median, IQR)  Fat-free mass (kg, mean, SD, median, IQR)  Fat mass (kg, mean, SD, median, IQR)  Missing | 58.8 (11.5, 56.2, 50.2-63.8)  44.1 (8.3, 42.9, 37.6-45.0)  32.8 (13.7, 30.9, 24.6-42.9)  11 |
| Resting Metabolic Rate (kcal/day, mean, SD, median, IQR)  Missing | 1306 (224, 1252, 1134-1420)  11 |
| Baseline Frailty (n)  Non-Frail  Pre-Frail  Frail  Missing | 7  18  7  3 |
| 1-Year Frailty (n)  Non-Frail  Pre-Frail  Frail  Missing | 4  6  3  22 |
| Attrition & Delays (n)  Unwilling to complete baseline evaluation (unknown reason)  1-Year follow-up delayed  Moved  Lost to follow-up (unknown reason)  Too physically sick to participate  Withdrawn or dropped out due to cognitive impairment  No longer interested  Afraid to return due to COVID-19 pandemic  Died  Total | 1  4  1  1  2  2  5  2  2  20 |

**Supplemental Table 2.** Adapted frailty phenotype criteria

|  | **Measure** | **Criteria Cut-Points** |
| --- | --- | --- |
| Unintentional weight loss | Current weight (measured) – self-reported weight 1 year prior | ≥ 5 percent of body weight or ten pounds lost |
| Weakness | Average of 3 dominant grip strength measurements (Jamar hydraulic dynamometer) and BMI | Men:  If BMI is ≤ 24, cut point is ≤ 29 kg  If BMI is 24.1-26, cut point is ≤ 30 kg  If BMI is 26.1-28, cut point is ≤ 30 kg  If BMI is >28, cut point is ≤ 32 kg    Women:  If BMI is ≤ 23, cut point is ≤ 17 kg  If BMI is 23.1-26, cut point is ≤17.3 kg  If BMI is 26.1-29, cut point is ≤18 kg  If BMI is > 29, cut point is ≤ 21 kg |
| Slow gait | Average of 3, 15-foot usual walks | Men:  If height is ≤ 173 cm (68.1 in), cut point is ≥ 7 seconds.  If height is > 173cm, cut point is ≥ 6 seconds.    Women:  If height is ≤ 159cm (62.6 in), cut point is ≥ 7 seconds.  If height is > 159cm, cut point is ≥ 6 seconds. |
| Low activity | 6-item Minnesota Leisure Time Physical Activity Questionnaire | Men: < 148 kcal/week    Women: < 105 kcal/week |
| Exhaustion | In the last week, how often did you feel that everything you did was an effort?    In the last week, how often did you feel that you could not get going? | Reported frequency ≥3-4 days (or a moderate amount of the time) in the prior 1 week to either question. |

**Supplemental Table 3: Ordinal Logistic Regression Model Relating 1-Year Frailty Phenotype (0-5) to Baseline RMR Quartiles in the Total Sample**

|  | Total Sample (n=116) |
| --- | --- |
| Independent Variables | Odds Ratio, (p value) |
| Resting Metabolic Rate Quartiles (kcal/day)  Lowest quartile  Second quartile  Third quartile  Highest quartile | ref  2.55 (0.10)  3.25 (0.07)  6.42 (0.02) |
| Frailty score (baseline) | 3.08 (<0.001) |
| Fat-Free Mass (per 1 kg mass) | 0.92 (0.06) |
| Fat Mass (per 1 kg mass) | 1.05 (0.05) |
| Race  Black  Other | 1. (0.94)   ref |
| Age | 1.06 (0.10) |
| Gender  Female  Male | 0.23 (0.09)  ref |
| Education  ≥High School  <High School | 0.20 (0.31)  ref |
| Monthly Income  <$2000  $2000-3999  $4000+/month  $6000+  Missing | ref  0.86 (0.73)  0.19 (0.02)  3.18 (0.14)  4.68 (0.15) |

**Supplemental Table 4: Ordinal Logistic Regression Model Relating 1-Year Frailty Phenotype to Baseline Resting Metabolic Rate Including Self-Reported Physical Activity Energy Expenditure as a Covariate (n=116)**

|  | Model 1 | Model 2 | Model 3 |
| --- | --- | --- | --- |
| Independent variables | Odds Ratio, (p value) | Odds Ratio, (p value) | Odds Ratio, (p value) |
| Resting Metabolic Rate (per 1 kcal/day) | 1.006 (0.001) | 1.006 (0.001) | 1.006 (0.001) |
| Frailty score (baseline) | 3.51 (<0.001) | 3.93 (<0.001) | 3.57 (<0.001) |
| Fat-Free Mass (per 1 kg mass) | 0.88 (0.01) | 0.88 (0.01) | 0.88 (0.01) |
| Fat Mass (per 1 kg mass) | 1.04 (0.14) | 1.03 (0.25) | 1.04 (0.15) |
| Average Weekly Physical Activity Energy Expenditure (per 1 kcal/week) | 1.0 (0.66) | 1.0 (0.75) | 1.0 (0.75) |
| Race  Black  Other | 1.01 (0.99)  Ref | 1.23 (0.74)  Ref | 1.09 (0.89)  Ref |
| Age (per year) | 1.06 (0.09) | 1.07 (0.08) | 1.06 (0.09) |
| Gender  Female  Male | 0.22 (0.09)  Ref | 0.20 (0.08)  Ref | 0.22 (0.09)  Ref |
| Education  ≥High School  <High School | 0.19 (0.28)  Ref | 0.19 (0.32)  Ref | 0.20 (0.30)  ref |
| Monthly Income  <$2000  $2000-3999  $4000-5999  $6000+  Missing | ref  0.80 (0.60)  0.15 (0.009)  2.86 (0.19)  5.71 (0.11) | ref  0.69 (0.40)  0.11 (0.004)  3.14 (0.16)  4.13 (0.20) | ref  0.81 (0.62)  0.13 (0.007)  2.91 (0.18)  5.23 (0.13) |
| Charlson Comorbidity Index | -- | 0.81 (0.16) | -- |
| Polypharmacy  <5 medications  ≧5 medications  Missing | -- | ref  1.75 (0.22)  1.96 (0.50) | -- |
| Montreal Cognitive Assessment Score | -- | -- | 1.07 (0.37) |

**Supplemental Table 5. Logistic Regression Relating 1-Year Frailty Phenotype Decline (1=Any Decline vs 0=Same/Better Score) to Baseline Resting Metabolic Rate**

| Independent variables | Odds Ratio, (p value) | |
| --- | --- | --- |
|  | Non-frail at baseline (n=46) | Pre-frail at baseline (n=62^2^) |
| Resting Metabolic Rate (per 1 kcal/day) | 1.003 (0.32) | 1.009 (0.02) |
| Frailty score (baseline) | -- (0 points) | 1.90 (0.38) (2 vs 1 point) |
| Fat-Free Mass (per 1 kg mass) | 0.92 (0.36) | 0.93 (0.49) |
| Fat Mass (per 1 kg mass) | 1.07 (0.17) | 1.00 (1.00) |
| Race  Black  Other | 1.21 (0.94)  Ref | 0.17 (0.13)  Ref |
| Age (per year) | 1.21 (0.02) | 1.03 0.74) |
| Gender  Female  Male | 0.21 (0.42)  Ref | 1.90 (0.74)  Ref |
| Education^1^  ≥High School  <High School | --  -- | 0.13 (0.23)  Ref |
| Monthly Income^1^  <$2000  $2000-3999  $4000+  Missing | Ref  1.71 (0.54)  0.77 (0.78)  9.71 (0.21)) | Ref  1.25 (0.79)  0.37 (0.44)  -- |

^1^Among the n=46 older adults who were non-frail at baseline, only n=1 had an education <High school, therefore the education variable was not included in the non-frail model. Due to small cell sizes, the $4000-5999/month and $6000+/month income categories were collapsed for both models. These adjustments were made to optimize model fitting and did not substantially change any independent variable effect sizes or level of statistical significance.

^2^Among the n=62 pre-frail at baseline, n=1 observation was dropped from this model due to lack of a comparison group for Missing income among those with worse 1-year frailty.
